# Molecular basis of carbapenem and colistin resistance and *phoQ* mutation-mediated colistin resistance in *Acinetobacter baumannii* from Bangladesh

**DOI:** 10.1128/spectrum.03601-25

**Published:** 2025-12-29

**Authors:** Pompy Dey, Sudip Kumar Chowdhury, Swarna Paul, Ayesha Ahmed Khan, Md. Fourkan Uddin, Milupa Nasrin, Fouzia Ahmed Chowdhury, Swagata Nandy, Abul Kalam

**Affiliations:** 1 Department of Microbiology, Rangamati Medical College, Rangamati, Bangladesh; 2 Department of Community Medicine and Public Health, Rangamati Medical College, Rangamati, Bangladesh; 3 Department of Microbiology, Chittagong Medical College 467859, Chattogram, Bangladesh; 4 Department of Microbiology, Institute of Applied Health Sciences 185965, Chattogram, Bangladesh; 5 Department of Microbiology, Gopalganj Medical College, Gopalganj, Bangladesh; 6 Department of Microbiology, Southern Medical College 469195, Chattogram, Bangladesh; 7 Department of Microbiology, Chattogram International Dental College, Chattogram, Bangladesh; Sacramento County Public Health Laboratory, Sacramento, California, USA

**Keywords:** *Acinetobacter baumannii*, *blaNDM-1*, carbapenem resistance, colistin resistance, molecular epidemiology, *phoQ* mutation

## Abstract

Carbapenem- and colistin-resistant *Acinetobacter baumannii* pose a serious global health threat due to limited therapeutic options and rapid dissemination of resistance determinants. This cross-sectional study investigated known carbapenemase and colistin resistance genes among clinical *A. baumannii* isolates from hospitalized patients in Chattogram, Bangladesh. A total of 325 clinical specimens (urine, wound swab, sputum, blood, and endotracheal aspirate) were processed by standard microbiological methods. Species confirmation was performed by 16S rRNA gene amplification. Antimicrobial susceptibility was determined by the Kirby-Bauer method, and colistin MICs were measured by broth microdilution. Among 239 bacterial isolates, 26 (10.9%) were confirmed as *A. baumannii*. High resistance rates were observed for ciprofloxacin (65.4%), cefepime (65.4%), meropenem (53.9%), and imipenem (46.2%). Four isolates (15.4%) were colistin-resistant, with MICs ranging from ≤0.5 to 64 µg/mL. Carbapenemase genes were detected in 16 (61.5%) isolates, predominantly *blaNDM-1* (50%), followed by *blaVIM* (7.7%) and *blaOXA-48* (3.8%), showing a strong association with carbapenem resistance (*P* = 0.0000124). The *phoQ* gene was identified in 7 (26.9%) isolates, with co-existence of *pmrC* in 2; four of these were colistin-resistant (*P* = 0.0023). Sequencing of *phoQ* revealed point mutations consistent with known resistance-associated variants. The predominance of *blaNDM-1* & *phoQ*-mediated resistance highlights the urgent need for molecular surveillance and stringent antimicrobial stewardship to curb the spread of multidrug-resistant *A. baumannii* in Bangladeshi hospitals.

**IMPORTANCE:**

Antibiotic-resistant *Acinetobacter baumannii* is a serious threat to patient health worldwide because it can resist many of the strongest available antibiotics, making infections difficult to treat. This study identifies the genes that allow these bacteria to survive last-resort drugs such as carbapenems and colistin in hospitals in Chattogram, Bangladesh. By pinpointing the common carbapenem-resistance gene (blaNDM-1) and colistin-resistance mutations (phoQ), the research shows how these bacteria evade treatment and highlights the risk of their spread in healthcare settings. Understanding these resistance mechanisms helps doctors choose effective therapies and informs hospital policies to prevent outbreaks. The findings underscore the urgent need for ongoing monitoring of antibiotic resistance and stronger stewardship of antibiotic use to protect patients and curb the rise of these dangerous “superbugs.”

## INTRODUCTION

Antimicrobial resistance (AMR) has become one of the most pressing global health challenges, as many bacterial pathogens now exhibit reduced susceptibility to existing drugs. Multidrug-resistant (MDR) Gram-negative ‘‘superbugs’’ such as *Acinetobacter baumannii*, *Klebsiella pneumoniae*, and *Pseudomonas aeruginosa* have emerged as major threats in healthcare settings worldwide (1). Among them, *A. baumannii* is particularly notorious due to its environmental persistence and remarkable genetic adaptability, which complicate infection control and treatment (2). It causes a broad range of nosocomial infections, including ventilator-associated pneumonia, bacteremia, urinary tract infections, and surgical-site infections (3).

Carbapenems, a subclass of β-lactam antibiotics, are widely regarded as last-line agents for severe infections caused by MDR Gram-negative bacteria because of their broad-spectrum bactericidal activity (4). Like other β-lactams, they act by binding to penicillin-binding proteins (PBPs), thereby inhibiting bacterial cell-wall synthesis and causing cell lysis. However, the clinical efficacy of carbapenems has been undermined by multiple resistance mechanisms, including reduced membrane permeability, overexpression of efflux pumps, alterations in PBPs, and, most importantly, carbapenemase production (5).

Carbapenemase enzymes that hydrolyze β-lactam antibiotics are classified into Ambler classes A (e.g., KPC), B (metallo-β-lactamases [MBLs] such as IMP, VIM, NDM), and D (oxacillinases, e.g., OXA-23 to OXA-27) (6). Among them, MBLs are zinc-dependent enzymes that can hydrolyze nearly all β-lactams except monobactams and are inhibited by metal chelators such as EDTA (7, 8). The first NDM-1 enzyme was reported in 2008 in a Swedish patient who had acquired an infection in India (9). Since then, plasmid-mediated dissemination of *blaNDM* and other MBL genes has been documented globally, accelerating the spread of carbapenem resistance among Gram-negative pathogens (10). Alarmingly high rates of carbapenem-resistant *A. baumannii* have been reported in South Asia, including 93.3% in Bangladesh and 14.3% in India (11, 12).

Colistin (polymyxin E), a polycationic peptide antibiotic first isolated from *Bacillus polymyxa* in 1947, was initially abandoned due to its nephrotoxicity and neurotoxicity (13). However, its use has resurged as a last-resort therapy against MDR, extensively drug-resistant (XDR), and pan–drug-resistant (PDR) Gram-negative bacteria (14, 15). Colistin interacts electrostatically with the lipid-A component of lipopolysaccharide (LPS) in the bacterial outer membrane, replacing divalent cations (Ca²^+^, Mg²^+^), leading to disruption of membrane integrity and bacterial lysis (16, 17). Nevertheless, the widespread use of colistin in both human and veterinary medicine, especially in livestock production in developing countries, has contributed to the emergence of colistin resistance (18). Recent studies have highlighted that resistance mechanisms, such as modifications in the phoQ regulatory system and the acquisition of resistance genes like blaNDM, further complicate treatment options. Addressing these challenges requires not only robust surveillance but also the exploration of alternative management strategies. A study emphasizes the importance of integrating phytomedicinal approaches alongside conventional therapies to combat antibiotic-resistant microbes (ARM). Their work underscores the potential of plant-derived compounds to inhibit resistance mechanisms and restore antibiotic efficacy, thereby providing a complementary strategy to current antimicrobial stewardship efforts (19).

Resistance to colistin arises through both chromosomal mutations and plasmid-mediated mechanisms. The plasmid-borne *mcr-1* gene encodes a phosphoethanolamine transferase that modifies lipid A, reducing the affinity of colistin for the bacterial membrane (20). Chromosomal mutations involving the two-component regulatory systems *pmrA/pmrB* & *phoP/phoQ*, as well as alterations in *mgrB*, *lpxC*, and *lpxD* genes, lead to modification of LPS via addition of 4-amino-4-deoxy-L-arabinose (L-Ara4N) or phosphoethanolamine groups, thereby decreasing the negative charge of lipid A and impairing colistin binding (21, 22). The *phoP/phoQ* system may also activate *pmrA/pmrB* through the *pmrD* linker protein, further promoting resistance (21).

Moreover, colistin-resistance genes are frequently located on mobile genetic elements, integrons, and transposons, enhancing their horizontal transfer among Gram-negative bacteria (23, 24). In Bangladesh, *A. baumannii* isolates from hospitalized patients demonstrated a 7.14% rate of colistin resistance, with *pmrA*, *pmrB*, and *phoP* being the most prevalent resistance genes; no plasmid-mediated *mcr-1* gene was detected (25, 26).

Given the clinical importance of *A. baumannii* and the growing prevalence of carbapenem- and colistin-resistance, continuous molecular surveillance is essential to guide effective antimicrobial stewardship. Therefore, the present study aimed to determine the antimicrobial susceptibility profile of *A. baumannii* isolates and to identify the prevalence of carbapenemase genes (*blaIMP*, *blaVIM*, *blaNDM-1*, *blaKPC*, *blaOXA-48*) using multiplex PCR. Additionally, colistin minimum inhibitory concentrations (MICs) were determined by broth microdilution, and resistance-associated genes (*phoP*, *phoQ*, *pmrA*, *pmrB*, *pmrC*, *mcr-1*) were analyzed. The *phoQ* gene was further sequenced to detect potential mutations relative to reference sequences in the NCBI GenBank database.

## RESULTS

### Distribution of *Acinetobacter baumannii* isolates

**Fig 1:**
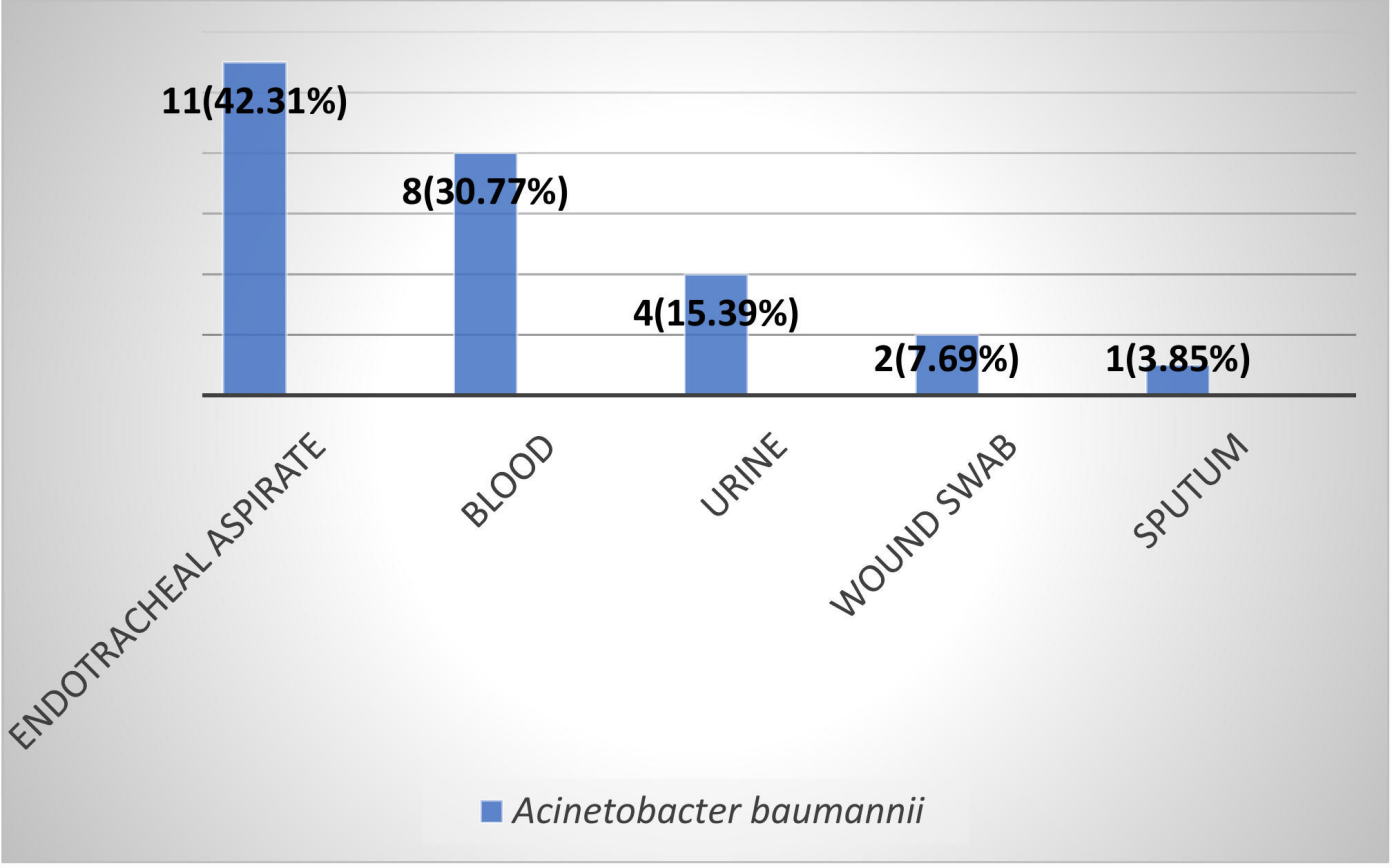
Distribution of *Acinetobacter baumannii* among different specimen (*n* = 26).

Among 325 clinical samples analyzed, 99 (30.46%) were urine, 87 (26.77%) wound swabs, 55 (16.92%) sputum, 45 (13.85%) blood, and 39 (12.00%) endotracheal aspirates. A total of 27 (11.30%) *Acinetobacter* spp. was identified among 239 bacterial isolates, of which 26 (10.88%) were confirmed as *A. baumannii* by PCR targeting the species-specific 16S rRNA gene. The distribution of *A. baumannii* among different specimen types is shown in Fig. 1.

### Antimicrobial susceptibility pattern

**Fig 2:**
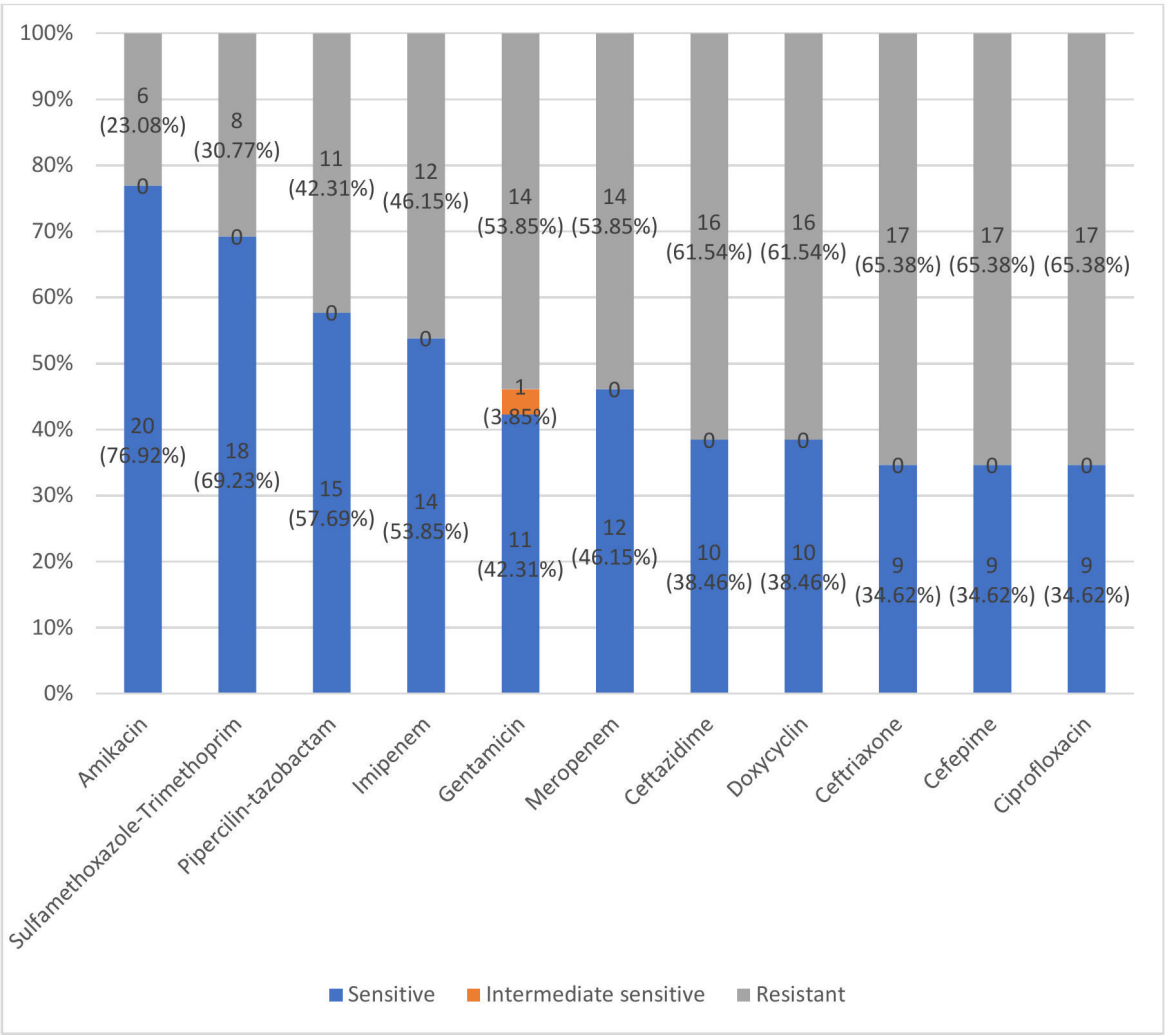
Antimicrobial susceptibility pattern of isolated *Acinetobacter baumannii* (*n* = 26).

Among the 26 *A*. *baumannii* isolates, resistance was observed to ciprofloxacin (65.38%), cefepime (65.38%), ceftriaxone (65.38%), ceftazidime (61.54%), doxycycline (61.54%), gentamicin (53.85%), meropenem (53.85%), imipenem (46.15%), piperacillin-tazobactam (42.31%), sulfamethoxazole-trimethoprim (30.77%), and amikacin (23.08%) (Fig. 2)

### Colistin susceptibility (MIC determination)

Colistin MIC values ranged from ≤0.5 µg/mL to 64 µg/mL. According to CLSI (27) interpretive criteria, 4 (15.38%) isolates were colistin-resistant (MIC ≥ 4 µg/mL), 22 (84.62%) were intermediate (MIC ≤ 2 µg/mL) sensitive (Table 1).

**TABLE 1:** MIC of colistin among *Acinetobacter baumannii* isolates (*n* = 26)

MIC (µg/mL)	*n* (%)	Susceptibility pattern
≥64	1 (3.84)	Resistant
32	0	
16	2 (7.69)	
8	0	
4	1 (3.85)	
2	5 (19.23)	Intermediate sensitive
1	15 (57.69)	
≤0.5	2 (7.69)	

### Molecular detection of carbapenemase genes

Carbapenemase-encoding genes were detected in 16 (61.54%) isolates by multiplex PCR. The primers used for specific genes included *blaNDM-1*, *blaVIM*, *blaIMP*, *blaKPC*, and *blaOXA-48*, with no co-existence of multiple carbapenemase genes in any isolate. Among the isolated *A. baumannii*, 13 (50%) harbored NDM-1, 2 (7.69%) VIM, and 1 (3.85%) OXA-48. Neither IMP nor KPC was found in these isolates. Among 14 (53.85%) carbapenem-resistant isolates (by disc diffusion), all were PCR-positive for carbapenemase genes, whereas only 2 (7.69%) of the 12 carbapenem-sensitive isolates were PCR-positive. The association was statistically significant (Fisher’s Exact test, *P* = 0.0000124) (Table 2)

**TABLE 2:** Association between phenotypic and genotypic detection of carbapenem resistance among *A. baumannii* (*n* = 26)

Phenotypic result	PCR positive *n* (%)	PCR negative *n* (%)	*P* value
Carbapenem-resistant	14 (53.85)	0	<0.0001
Carbapenem-sensitive	2 (7.69)	10 (38.46)	
**Total**	**16 (61.54)**	**10 (38.46)**	

### Detection of colistin resistance genes

Colistin resistance genes were detected in 7 (26.92%) isolates, all carrying *phoQ* with co-existence of *pmrC* gene in two isolates. No *phoP, pmrA, pmrB,* & *mcr-1* were observed. Among 4 (15.38%) colistin-resistant isolates by broth microdilution, all were PCR-positive for *phoQ*, whereas only 3 (11.54%) of 22 intermediate isolates carried *phoQ* and 2 (7.69%) of them co-existed with carrying *pmrC*. The difference was statistically significant (Fisher’s Exact test, *P* = 0.0023) (Table 3).

**TABLE 3:** Comparison between phenotypic and genotypic colistin resistance among *A. baumannii* (*n* = 26)

Colistin susceptibility	PCR positive *n* (%)	PCR negative *n* (%)	*P* value
Resistant	4 (15.38)	0	<0.05
Intermediate-sensitive	3 (11.54)	19 (73.08)	
**Total**	**7 (26.92)**	**19 (73.08)**	

### Phenotypic and genotypic characterization of carbapenem and colistin resistance among phoQ-harbored *A. baumannii* isolates

Among seven *A. baumannii* isolates, five (71.4%) were resistant to both imipenem and meropenem. The predominant carbapenemase gene detected was *blaNDM-1* (5/7), followed by *blaOXA-48* & *blaVIM* (1/7 each). Colistin MICs ranged from 2 to 64 µg/mL, with four isolates (57.1%) showing phenotypic resistance (MIC ≥ 4 µg/mL). All phenotypically detected colistin resistant isolates harbored *phoQ*, while pmrC was co-detected in two isolates (p64 and p232) (Table 4).

**TABLE 4:** Carbapenem susceptibility, distribution of carbapenemase genes, MIC values of colistin, and colistin susceptibility among colistin resistance gene-detected isolates (*n* = 7)

Sample ID	Carbapenem susceptibility		Detected carbapenemase gene	MIC of colistin (µg/mL)	Colistin susceptibility	Colistin resistant gene
	Imipenem	Meropenem				
p52	Resistant	Resistant	*blaNDM-1*	4	Resistant	*phoQ*
p64	Resistant	Resistant	*blaNDM-1*	16	Resistant	*phoQ, pmrC*
p139	Sensitive	Resistant	*blaOXA-48*	2	Intermediate sensitive	*phoQ*
p141	Sensitive	Sensitive	*blaVIM*	2	Intermediate sensitive	*phoQ*
p162	Resistant	Resistant	*blaNDM-1*	2	Intermediate sensitive	*phoQ*
p232	Resistant	Resistant	*blaNDM-1*	64	Resistant	*phoQ, pmrC*
p260	Sensitive	Resistant	*blaNDM-1*	16	Resistant	*phoQ*

Four phenotypically detected colistin resistant isolates were positive for presence of mutated *phoQ* gene, which were confirmed by automated Sanger sequencing. Those sequences were submitted to NCBI Genbank database and the given accession numbers are PX418253.1 (Sample ID: p260), PX418254.1 (Sample ID: p64), PX418255.1 (Sample ID: p52), and PX418256.1 (Sample ID: p232).

**Fig 3:**
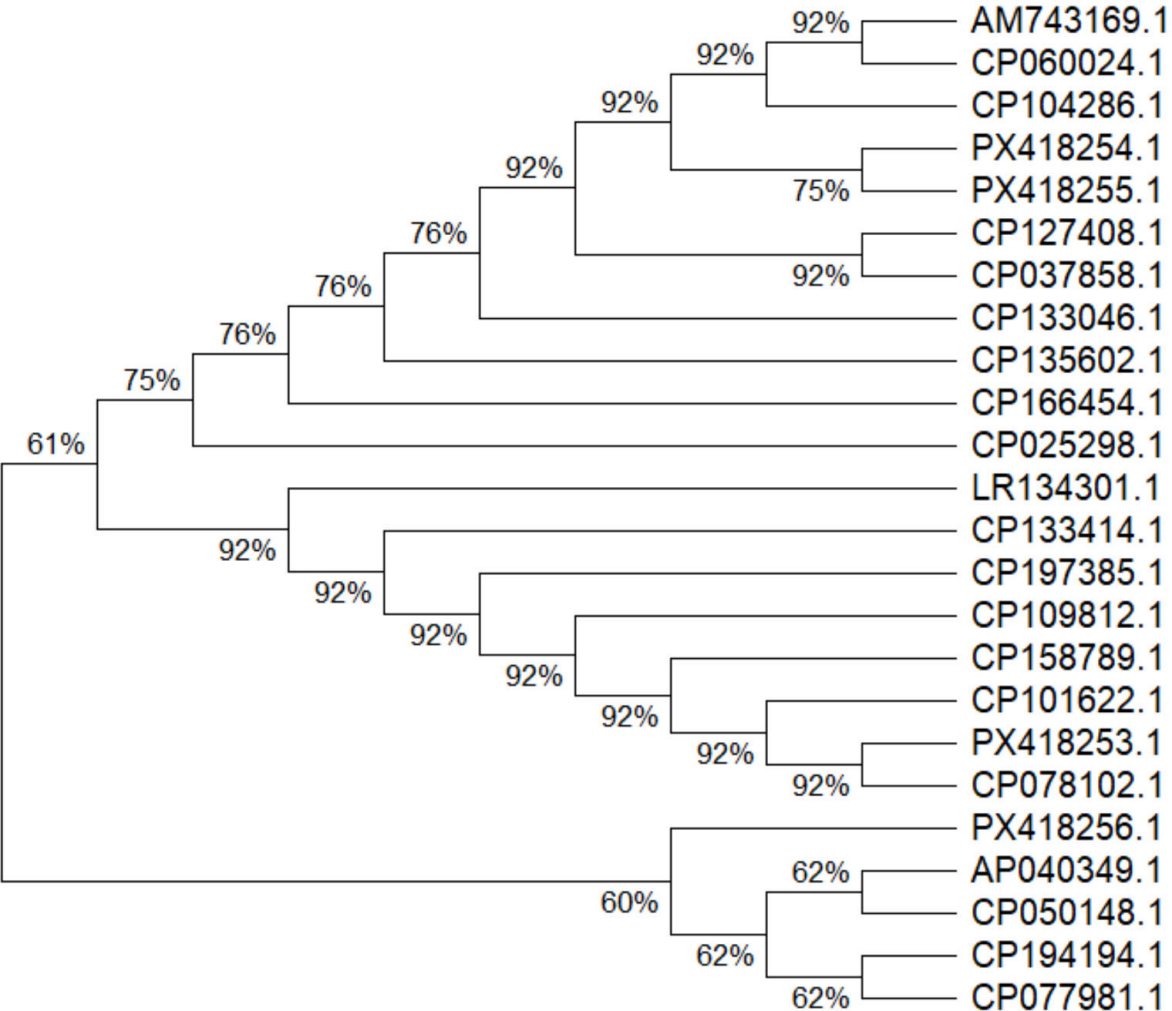
Phylogenetic tree of 24 sequences by N-J method.

The phylogenetic tree was constructed with the four sequences along with 20 other sequences retrived from NCBI genbank. The evolutionary history was inferred using the Neighbor-Joining method (28). The optimal tree with the sum of branch length = 5.566 is shown. The percentage of replicate trees in which the associated taxa clustered together in the bootstrap test (1,000 replicates) is shown (29). The evolutionary distances were computed using the Maximum Composite Likelihood method (30) and are in the units of the number of base substitutions per site. Evolutionary analyses were conducted in MEGA12 (31) (Fig. 3).

## DISCUSSION

In this study, *Acinetobacter* spp. accounted for 11.30% (27/239) of all bacterial isolates, with 26 (10.88%) confirmed as *A. baumannii* by 16S rRNA PCR. This prevalence is comparable to findings from Russia and Kazakhstan (10.2%) (32) and Ethiopia (9.13%) (33), but higher than the pooled prevalence of 3.99% reported in a systematic review (33). Global analyses indicate rising *A. baumannii* prevalence, particularly in Asia and the Western Pacific (34). PCR-based 16S rRNA identification remains reliable (35, 36) though use of additional targets such as *rpoB*r*blaOXA-51-like* can enhance specificity (32, 37).

High resistance rates were observed to ciprofloxacin (65.38%), cephalosporins (61.54-65.38%), doxycycline (61.54%), and carbapenems (46.15%–53.85%), reflecting the global burden of MDR *A. baumannii*. These rates parallel global averages for imipenem (44.7%) and meropenem (59.4%) resistance (38) but are somewhat lower than in Southeast Asia, where resistance often exceeds 80%–90% (39–41). The comparatively low amikacin resistance (23.08%) may reflect differences in local antibiotic pressure or clonal variation. Overall, the resistance profile underscores the urgent need for antimicrobial stewardship and stringent infection control.

Colistin MICs ranged from ≤0.5 to 64 µg/mL, with 15.38% of isolates resistant, 76.92% intermediate, and 7.69% susceptible. All resistant isolates were from endotracheal aspirates, suggesting an association with respiratory infections. Although global colistin resistance averages 1%–7% (39, 42–44), higher rates (>20%) have been reported in ICUs and high antibiotic-use settings (45). Similar trends have been observed in Asia, with ICU isolates showing resistance between 5% and 20% (46, 47). Mechanisms typically involve chromosomal mutations in *pmrCAB* or *phoPQ* operons and lipid A modification (48, 49). The presence of colistin-resistant strains exclusively in respiratory samples aligns with reports linking such isolates to biofilm formation on medical devices (46, 47).

Carbapenemase genes were detected in 61.54% of isolates, predominantly *blaNDM-1* (50%), followed by *blaVIM* (7.69%) and *blaOXA-48* (3.85%), consistent with regional reports (50, 51). The absence of *blaIMP* & *blaKPC* mirrors findings from other Asian studies (52). All phenotypically carbapenem-resistant isolates were PCR-positive for carbapenemase genes, confirming their key role in resistance. Unlike reports from India and China, no co-existence of multiple carbapenemase genes was found, possibly reflecting local epidemiological differences (50, 51).

Colistin resistance genes were identified in 26.92% of isolates, all harboring *phoQ*, with *pmrC* co-expressed in two. The significant association between colistin resistance genes detection in *phoQ* and phenotypic resistance (*P* = 0.0023) supports previous evidence implicating *phoQ/pmrC*-mediated lipid A modification in colistin resistance (48, 53). The absence of *mcr-1* and other *pmr* genes (other than *pmrC*) suggests chromosomal mechanisms rather than plasmid-mediated transfer. Although *pmrCAB* mutations are frequently implicated in colistin resistance, several studies show that many missense variants in this operon occur in both resistant and susceptible isolates, indicating that they may represent neutral, lineage-specific polymorphisms rather than true resistance determinants. Therefore, the lack of *pmrCAB* mutations in our isolates may simply reflect the absence of functionally relevant substitutions rather than the absence of resistance (43, 54).

Colistin resistance in *A. baumannii* can also arise through alternative pathways. Overexpression of *eptA*, often driven by insertion elements such as ISAba1, may mediate resistance independently of *pmrCAB* changes. Additionally, phage-associated acquisition of lipid A-modifying genes (e.g., *eptA1*) has been reported, further decoupling resistance from *pmrCAB* mutations (49, 54).

Geographic and clonal variability in resistance mechanisms may also explain differences across studies, as some lineages rely more heavily on non-*pmrCAB* pathways (54). Finally, mutation-based screening may miss novel or regulatory alterations affecting gene expression, which can confer resistance without detectable coding-region mutations (43, 54).

While the dominance of phoQ mutations, confirmed by Sanger sequencing and phylogenetic analysis, highlights its evolutionary significance in resistance development (55, 56), it is important to contextualize these findings within the broader landscape of molecular methodologies. Our PCR-based approach, while effective for targeted mutation detection, is inherently limited in scope compared to whole-genome sequencing (WGS) and in-silico resistance gene prediction models. WGS enables comprehensive profiling of all genetic determinants of resistance, including novel or unexpected mutations, and facilitates high-resolution epidemiological tracking (57). *In silico* prediction tools, as demonstrated in some studies, leverage WGS data to accurately predict antibiogram profiles by identifying both known and acquired resistance genes, thus providing a more holistic view of resistance mechanisms (57).

By contrast, PCR and Sanger sequencing are restricted to predefined targets and may overlook additional resistance determinants or genetic contexts influencing resistance phenotypes. This methodological gap underscores the need for integrating WGS and advanced bioinformatics pipelines into routine surveillance, as they offer superior sensitivity, specificity, and scalability for monitoring resistance evolution (57). Our focused analysis adds to the current understanding of A. baumannii molecular epidemiology but also highlights the necessity for genomic surveillance using state-of-the-art approaches to fully capture the complexity of resistance development (56, 58).

>*Acinetobacter baumannii* is prevalent in this tertiary care hospital in Bangladesh, exhibiting high carbapenem resistance, mainly mediated by *blaNDM-1*, and emerging colistin resistance driven by *phoQ* mutations, with strong concordance between phenotypic and genotypic profiles. The absence of PFGE and MLST analyses prevents the determination of whether the observed 15.4% colistin resistance reflects spontaneous emergence or a clonal outbreak. The limited sample size (26 isolates from 325 specimens) constrains generalization regarding the molecular epidemiology of the region. Although urine samples accounted for the majority of specimens (30.46%), specific resistance genes such as *qnr*, which often co-occur in urinary bacterial consortia, were not screened, restricting conclusions about multidrug resistance in urinary isolates (59). These findings emphasize the need for routine molecular surveillance, robust antimicrobial stewardship, and larger multicenter genomic studies to better understand resistance mechanisms, dynamics, and clonal dissemination.

## MATERIALS AND METHODS

### Study design and setting

A cross-sectional study was conducted in the Department of Microbiology, Chittagong Medical College, Bangladesh, from July 2022 to June 2023. A total of 325 clinical specimens (urine, wound swab, sputum, blood, and endotracheal aspirate) were collected from admitted patients in various departments of Chittagong Medical College Hospital after obtaining informed written consent.

### Eligibility criteria

Patients from all age groups and genders with suspected bacterial infections were included. Samples from patients or guardians refusing consent were excluded. Demographic and clinical information was collected using a structured data sheet.

### Culture and identification

Samples were processed within 2 h of collection according to standard microbiological techniques (60, 61). Semi-quantitative cultures were performed using a calibrated loop on blood agar and MacConkey agar plates and incubated at 37°C for 24 h. Blood specimens were processed using the BD BACTEC FX40 automated culture system, followed by subculture. *Acinetobacter* isolates were identified by colony morphology, Gram staining, oxidase and catalase testing, and motility. Confirmation of *A. baumannii* was performed by PCR targeting the 16S rRNA gene.

### Antimicrobial susceptibility testing

Antimicrobial susceptibility testing (AST) was performed by the modified Kirby–Bauer disc diffusion method on Mueller–Hinton agar and interpreted according to CLSI guidelines (27). The antibiotics tested included amikacin, cefepime, ceftazidime, ceftriaxone, ciprofloxacin, doxycycline, gentamicin, imipenem, meropenem, piperacillin-tazobactam, and sulfamethoxazole-trimethoprim (Oxoid, UK).

Colistin susceptibility was determined by the broth microdilution (BMD) method following CLSI M07-A9 standards (62). *E. coli* ATCC 25922, *P. aeruginosa* ATCC 27853, and *S. aureus* ATCC 25923 were used as quality control strains.

### Molecular detection of resistance genes

Genomic DNA was extracted using the Monarch Genomic DNA Purification Kit (New England Biolabs, USA) according to the manufacturer’s protocol. Multiplex PCR assays were conducted to detect carbapenemase genes (*blaNDM-1*, *blaVIM*, *blaKPC*, *blaOXA-48*, and *blaIMP*) and colistin resistance genes (*phoP*, *phoQ*, *pmrA*, *pmrB*, *pmrC*, *mcr-1*).

Each 25 µL reaction mixture contained 12.5 µL of master mix, 1 µL of each primer (Table 5), 2 µL DNA template, and remaining µL of nuclease-free water. The PCR conditions were as follows:

- For 16S rRNA, *blaVIM*, *blaKPC*, *blaOXA-48*, and *mcr-1*: initial denaturation at 95°C for 3 min; 40 cycles of denaturation at 95°C for 1 min, annealing at 56°C for 40 s, and extension at 72°C for 1 min; final extension at 72°C for 5 min.
- For *phoP/Q, pmrA/B/C*, and *blaNDM-1*: initial denaturation at 95°C for 5 min; 36 cycles of denaturation at 95°C for 1 min, annealing at 57°C for 40 s, and extension at 72°C for 1 min; final extension at 72°C for 10 min.
- For *blaIMP*: initial denaturation at 95°C for 5 min; 36 cycles of denaturation at 95°C for 1 min, annealing at 54°C for 40 s, and extension at 72°C for 1 min; final extension at 72°C for 5 min.

**TABLE 5:** Primer sequences with product size

Target	Sequences (3′−5′)	Product length (bp)
16SrRNA	F-AGAGTTTGATCCTGCCTCAG R-TACCAGGGTATCTAATCCTGTT	790
blaIMP	F-GGAATAGAGTGGTGCTTAAYTCTC R-GGTTTAAYAAAACAACCACC	232
blaVIM	F-TGGCAACGTACGCATCACC R- CGCAGCACCGGGATAGAA	143
blaNDM-1	F- GCATTAGCCGCTGCATT R- GATCGCCAAACCGTTGG	100
blaKPC	F- CTGTATCGCCGTCTAGTTCTG R- AGTTTAGCGAATGGTTCCG	101
blaOXA-48	F-TTCCCAATAGCTTGATCGC R-CCATCCCACTTAAAGACTTGG	70
phoP	F-GAGCGTCAGACTACTATCGA R-GTTTTCCCATCTCGCCAGCA	912
phoQ	F-CCACAGGACGTCATCACCA R-GCAGGTGTCTGACAGGGATT	1594
pmrA	F-CGCAGGATAATCTGTTCTCCA R-GGTCCAGGTTTCAGTTGCAA	808
pmrB	F-GCGAAAAGATTGGCAAATCG R- GCGAAAAGATTGGCAAATCG	659
pmrC	F-CTCTCGCCTCGTTCCTGAA R-CGGAGTGGTGTCGAGGATA	140
mcr1	F-CGGTCAGTCCGTTTGTTTC R- CTTGGTCGGTCTGTAGGG	309

Amplicons were visualized on 2% agarose gels stained with SYBR Green under UV light, and band sizes were compared with a 100 bp DNA ladder.

### Sequencing and phylogenetic analysis

*phoQ* gene amplicons were purified and sequenced (Macrogen, Korea) using the same forward primers as in PCR. Sequence data were analyzed and trimmed with BioEdit v7.1.3. Phylogenetic trees were constructed using MEGA XII software with the neighbor-joining method, 1,000 bootstrap replicates, and the Kimura 2-parameter model. Sequence homology was confirmed through NCBI BLAST analysis.

### Data analysis

Data were compiled using Microsoft Excel and analyzed with SPSS version 27.0 (IBM, USA). Descriptive statistics were calculated, and inferential analyses were performed where appropriate. A *P*-value of <0.05 was considered statistically significant.

## Data Availability

The data sets used and/or analyzed during the current study are available from the corresponding author on reasonable request.
